# *Gastrodia elata* Blume extract improves high-fat diet-induced type 2 diabetes by regulating gut microbiota and bile acid profile

**DOI:** 10.3389/fmicb.2022.1091712

**Published:** 2022-12-02

**Authors:** Danqi Wang, Jun-Xia Wang, Chunri Yan, Yize Liu, Hongye Liu, Dongxu Li, Jun Zhu, Zhao-Bo Luo, Sheng-Zhong Han, Zheng-Yun Jin, Shuang-Yan Chang, Liu-Hui Yang, Jin-Dan Kang, Lin-Hu Quan

**Affiliations:** ^1^Interdisciplinary Program of Biological Functional Molecules, College of Integration Science, Yanbian University and Key Laboratory of Natural Medicines of the Changbai Mountain, Ministry of Education, College of Pharmacy, Yanbian University, Yanji, Jilin, China; ^2^Department of Animal Science, College of Agricultural, Yanbian University, Yanji, Jilin, China; ^3^Department of Preventive Medicine, Medical College, Yanbian University, Yanji, Jilin, China

**Keywords:** *Gastrodia elata* Blume, type 2 diabetes, insulin resistance, gut microbiota, bile acid

## Abstract

In this study, we aimed to characterize the anti-type 2 diabetes (T2D) effects of *Gastrodia elata* Blume extract (GEBE) and determine whether these are mediated through modification of the gut microbiota and bile acids. Mice were fed a high-fat diet (HFD), with or without GEBE, and we found that GEBE significantly ameliorated the HFD-induced hyperglycemia, insulin resistance, and inflammation by upregulating glucose transporter 4 (GLUT4) and inhibiting the toll-like receptor 4-nuclear factor kappa-B signaling pathway in white adipose tissue (WAT). In addition, we found that GEBE increased the abundance of *Faecalibaculum* and *Lactobacillus*, and altered the serum bile acid concentrations, with a significant increase in deoxycholic acid. The administration of combined antibiotics to mice to eliminate their intestinal microbiota caused a loss of the protective effects of GEBE. Taken together, these findings suggest that GEBE ameliorates T2D by increasing GLUT4 expression in WAT, remodeling the gut microbiota, and modifying serum bile acid concentrations.

## Introduction

With the gradual improvement in living standards and the associated consumption of a higher-energy diet and reduction in physical activity, type 2 diabetes (T2D) has become a highly prevalent disease (Shibib et al., 2022). T2D is a complex disease that is characterized by endocrine and metabolic defects, in which insulin resistance is a key pathogenic feature (Patrone et al., 2014). Insulin resistance is defined as poor sensitivity of insulin target tissues to insulin, which results in lower uptake and utilization of glucose (Minokoshi et al., 2004). Glucose transporter 4 (GLUT4) is the principal molecule responsible for insulin-stimulated glucose uptake by peripheral tissues, and it therefore plays an important role in systemic glucose homeostasis (Deng and Yan, 2016; Yuan et al., 2022).

The gut microbiota is now considered to be a key participant in the regulation of host metabolism and health (Quan et al., 2020; Xia et al., 2021), and recent studies have suggested that it plays an important role in the development of T2D (Zhao et al., 2018). Dietary supplementation with *Lactobacillus rhamnosus* GG results in increases in *Glut4* mRNA expression in muscle and adipose tissue, and an improvement in insulin sensitivity in high-fat diet (HFD)-fed mice (Nam et al., 2022). The roles of the gut microbiota in metabolic disorders are mainly determined by interactions between its metabolites, such as bile acids, fatty acids, and amino acids, with receptors on host cells (Wahlström et al., 2016). Primary bile acids, such as cholic acid (CA) and chenodeoxycholic acid (CDCA), are synthesized in the liver from cholesterol and then combined with taurine or glycine and stored in the gallbladder. After a meal, bile acids are expelled from the gallbladder into the intestine, where they are de-conjugated by bile salt hydrolase produced by the intestinal microbiota to form secondary bile acids (Wahlström et al., 2016). These bile acids can affect host metabolism by activating farnesoid X receptor (FXR) and Takeda G protein-coupled receptor 5 (TGR5) (Wahlström et al., 2016; Sun et al., 2018; Li et al., 2020; de Vos et al., 2022). For instance, glycoursodeoxycholic acid inhibits intestinal FXR signaling and increases the circulating concentration of glucagon-like peptide (GLP)-1, thereby improving glucose homeostasis in HFD-fed obese mice (Sun et al., 2018).

Traditional Chinese medicine (TCM) has been used for thousands of years and is effective for the treatment of systemic metabolic diseases, through the effects of multiple components on multiple targets (Zhang et al., 2021). Herbs such as *Ganoderma lucidum* reduce obesity and insulin resistance in obese mice by modulating the intestinal microbiota (Chang et al., 2015). In addition, *Hirsutella sinensis* ameliorates obesity, inflammation, and insulin resistance in HFD-fed mice by causing an enrichment of *Parabacteroides goldsteinii*, a bacterial species that lives exclusively in the gut (Wu et al., 2019). Finally, we previously found that ginseng extract increases the proportion of *Enterococcus faecalis* in the gut of mice and increases its production of myristic acid, which also reduces obesity (Quan et al., 2020). *Gastrodia elata* Blume (GEB) is a TCM that has been included in the diet and used for medicinal purposes (Lu et al., 2020). As one of the characteristic and main active components of GEB, gastrodin has been reported to reduce the ubiquitination of insulin receptors and improve insulin resistance (Bai et al., 2021). In addition, the polysaccharide component of GEB can produce beneficial effects by regulating the intestinal flora (Huo et al., 2021). GEB has antidepressant-like effects in a mouse model of depression that are mediated by alterations in the composition and function of the intestinal microbiota (Huang et al., 2021). However, it has not been determined whether the beneficial effects of GEB in T2D are exerted through modification of the structure of the intestinal flora and the range of metabolites produced.

In the present study, we administered GEB extract (GEBE) to HFD-fed mice to reveal its efficacy against T2D. We also evaluated the effects of GEBE on the composition of the gut microbiota and the host bile acid profile using 16S rRNA sequencing and a metabolomic approach, respectively. In addition, we administered antibiotics to eliminate the intestinal bacteria in mice to determine whether GEBE affects host glucose metabolism by altering the composition of the gut microbiota and the bile acids generated.

## Materials and methods

### Preparation of *Gastrodia elata* Blume extract

GEB with the product lot no. 190828009 (Beijing Qian Cao Traditional Chinese Medicine Co., Ltd., Beijing, China) was purchased from Beijing Tong Ren Tang Pharmacy in Yanji (Jilin, China). As described previously (Ng et al., 2016), dried GEB was powdered and extracted by heating reflux in distilled water twice for 1 h each. After filtration, the two filtrates obtained were combined and concentrated at atmospheric pressure to yield GEBE, which was diluted to the desired concentration prior to each gavage.

To assess the quality of GEBE, gastrodin was determined by high-performance liquid chromatography (HPLC) as it is the main bioactive compound of GEB. The GEBE and gastrodin standard (Shanghai Macklin Biochemical Technology Co., Ltd., Shanghai, China) were diluted to the desired concentrations and filtered through a 0.45-μm filter. Chromatographic analysis was performed using an Agilent 1,260 series liquid chromatography system (Agilent Technologies, California, USA) with a UV detector. Sample separation was achieved on an Agilent Poroshell ZORBAX SB-C18 (Agilent Technologies) column (4.6 mm × 150 mm, 5 μm) at a flow rate of 0.5 mL/min. The mobile phase solvent was 0.1% formic acid aqueous solution and acetonitrile (97:3, v/v) with isocratic elution. The injected sample volume was 5 μL and chromatograms were recorded at 270 nm. A calibration curve was prepared by measuring the peak area of a known gastrodin standard in the concentration range of 1–50 μg/mL. The linear regression equation for the calibration curve was *y* = 1.2756*x*–1.4011, (regression coefficient (*R*^2^ = 0.9974), where *x* is the concentration of gastrodin (μg/mL) and *y* is the peak area. The HPLC profile of gastrodin standard and GEBE are shown in Supplementary Figure 1, respectively. The concentration of gastrodin in our GEBE (1.00 mg/mL) was 3.92 μg/mL. The determined content of gastrodin in our extract was 0.392%, which is higher than the requirement of Chinese Pharmacopeia 2022 (0.25%).

The proximate compositions of crude protein, total fat, ash, crude fiber, and sodium (Na) of GEBE were analyzed according to the Chinese national standards, namely, GB 5009.5-2016, GB 5009.6-2016, GB 5009.4-2016, GB/T 5009.10-2003, and GB 5009.268-2016. And the content of crude polysaccharides was determined according to the Entry-Exit Inspection and Quarantine industry standards of China (SN/T 4260-2015).

### Animals

Mice were purchased from the Beijing Vital River Laboratory Animal Technology Co., Ltd. (Beijing, China). Four-week-old male C57BL/6 mice were housed under a 12-h light/dark cycle in a specific pathogen-free facility. Previously, it was reported that HFD with 60% fat supply induced obese T2D mice (Rozenberg and Rosenzweig, 2018). Therefore, we induced obese T2D mice using HFD according to our previous experimental approach (Quan et al., 2020). The mice were given free access to an HFD (60% kcal as fat; Beijing HuaFuKang Bioscience, Beijing, China) and water. To determine the effects of GEB on glucose metabolism, the gut microbiota, and the host bile acid profile, the mice were randomly allocated to two groups, an HFD group and a GEBE-treated group (HFD + GEBE), and gavaged with water or 200 mg/kg GEBE as previous study (Liu et al., 2021) at a designated time daily for 12 weeks. To study the role of the gut microbiota in the effects of GEBE in a mouse model of HFD-induced diabetes, we treated the mice with combined antibiotics to eliminate the gut bacteria. The mice were randomly allocated to two groups, an antibiotic treatment group (HFD + ABX) and a group that was also administered GEBE (HFD + ABX + GEBE). The mice were fed the HFD, and sterile water containing a combination of antibiotics [50 μg/mL streptomycin, 100 U/mL penicillin, 100 μg/mL metronidazole, 125 μg/mL ciprofloxacin, and 170 μg/mL gentamycin (all from Sigma Aldrich, St. Louis, MO)] was provided *ad libitum*. During the ABX treatment, the mice were administered water or GEBE by gavage each day for 12 weeks. At the end of this treatment period, the mice were euthanized after 16 h of fasting. Serum, white adipose tissue (WAT), brown adipose tissue (BAT), liver, and muscle samples were collected and immediately snap-frozen in liquid nitrogen for subsequent western blot analysis and other biochemical assays. The animal study was reviewed and approved by the Animal Ethics Committee of Yanbian University (SYXK2020-0009).

### Glucose and insulin tolerance testing

Glucose tolerance testing (GTT) was performed after 16 h of fasting. The glucose concentrations of blood obtained from a tail vein were measured using a glucometer (Yuyue, Jiangsu, China) 0, 15, 30, 60, 90, and 120 min after the intraperitoneal injection of 1.5 g/kg glucose (Sigma Aldrich). Insulin tolerance testing (ITT) was performed after a 4 h fast. An intraperitoneal injection of 0.75 U/kg insulin (Novo Nordisk, Bagsvaerd, Denmark) was administered and the glucose concentrations of blood from a tail vein were measured 0, 15, 30, 45, and 60 min later. Glucose and insulin tolerance were assessed using the respective areas under the glucose-time curves (AUCs).

### Physical activity and energy intake

A small animal activity recorder (SA-YLS-1C; Jiangsu Science Biological Technology Co. Ltd., Jiangsu, China) was used to record the movements of the mice. Prior to the start of the experiment, the mice were housed in individual cages for 24 h to acclimatize them to their new environment, and they were allowed to eat and drink freely throughout the experiment. The activity of the mice was measured over a 24 h period, and the mass of food provided at the beginning of the experiment and the mass left at the end were recorded to permit the calculation of the food intake of the mice. The feces of the mice were also collected, dried at 60°C, and weighed. A calorimeter (IKA C2000 basic; Staufen, Germany) was used to measure the energy contents of the food and the feces to calculate the energy expenditure of the mice.

### Western blot analysis

Tissue lysates were prepared in RIPA buffer (Beyotime, Shanghai, China) according to a previously described method (Luo et al., 2019). The protein concentration of each lysate was then measured using a BCA kit (Beyotime), and lysates containing equal amounts of protein were separated by SDS-PAGE (8–10%) electrophoresis and then transferred to PVDF membranes (Merck, Darmstadt, Germany). The membranes were blocked using 5% dried non-fat milk powder diluted in Tris-buffered saline containing 0.1% Tween-20 (TBS-T) and then incubated overnight at 4°C with the appropriate primary antibody. The next day, the membranes were incubated with the appropriate secondary antibody for 1 h. Specific bands were visualized using the GelView 6000Plus smart imaging system (Boluteng, Guangzhou, China). The antibodies used for western blotting were as follows: anti-β-actin (1:2,000, Bioss, Beijing, China), goat anti-rabbit secondary antibody (1:5,000, Bioss), goat anti-mouse secondary antibody (1:5,000, Beyotime), anti-GLUT4, anti-Toll-like receptor 4 (TLR4), and anti-nuclear factor kappa-B (NF-κB) (1:2,000, Cell Signaling Technology, Boston, MA).

### Immunohistochemistry

WAT samples were fixed in 10% formalin, dehydrated, and embedded in paraffin. Immunohistochemical staining was performed on paraffin sections as described previously (Qi et al., 2019). Briefly, sections were incubated with anti-GLUT4 antibody (Abcam, Cambridge, UK) overnight at 4°C, washed with PBS, and then incubated with secondary antibody (Beyotime) for 15 min at room temperature. DAB chromogenic solution (ZSGB-BIO, Beijing, China) was added and the sections were incubated for a further 5 min, counterstained with hematoxylin, dehydrated with increasing concentrations of ethanol and xylene, and then mounted. Images were obtained using an Olympus BX53 microscope (Olympus, Tokyo, Japan).

### Analysis of the gut microbiota

We collected fecal samples from the mice after 12 weeks of treatment for gut microbiological analysis, which was performed as previously described (Sun et al., 2020). Genomic DNA was extracted from the fecal samples using the CTAB method, and then the V3-V4 regions of the 16S rRNA genes were amplified using specific primers. The PCR products were separated on 2% agarose gels and purified using a Gel Extraction Kit (Qiagen, Hilden, Germany). Sequencing was then performed on an Illumina HiSeq (San Diego, CA), and the data obtained were processed and optimized for statistical analysis.

### Bile acid analysis

We collected serum samples for bile acid analysis prior to the euthanasia of the mice. The bile acids were extracted and analyzed as previously described (Zhou et al., 2015), using ice-cold methanol: acetonitrile (5:3) deuterated internal standards. The supernatants were dried in OH mode on a SpeedVac and resuspended in methanol prior to bile acid separation and analysis by ultra-high performance liquid chromatography-mass spectrometry (UPLC-MS). Each bile acid was quantified with reference to the result for the corresponding deuterated internal standard.

### Statistics

Data are expressed as the mean ± standard error of the mean (SEM) and Student’s *t*-test was used to compare the groups. The statistical significance level was set at *p < 0.05; ^**^p < 0.01; ^***^p < 0.001.

## Results

### *Gastrodia elata* Blume extract ameliorates the high-fat diet-induced inflammation and abnormal glucose tolerance

The proximate analysis of GEBE shows a content in protein of 1.29 g/100 g, total fat of 0.3 g/100 g, carbohydrates of 50.24 g/100 g, energy of 875.62 kJ/100 g, ash of 4.22 g/100 g, crude fiber of 2.48 g/100 g, sodium (Na) of 0.063 g/100 g, and crude polysaccharides of 30.8 g/100 g (Table 1).

**TABLE 1:** The components in GEBE.

Contents	Quantity (g/100 g)
Protein	1.29
Total fat	0.3
Carbohydrates	50.24
Energy (kJ)	875.62
Ash	4.22
Crude fiber	2.48
Sodium (Na)	0.063
Crude polysaccharides	30.8

**FIGURE 1:**
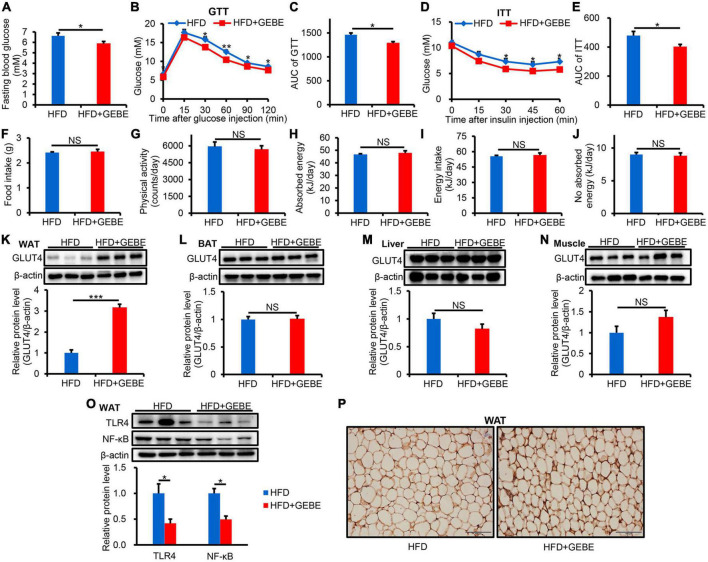
The effects of GEBE on glucose metabolism and inflammation in HFD-induced C57BL/6 mice. HFD-induced C57BL/6 mice were treated with GEBE (200 mg/kg) for 8 weeks by daily oral gavage (*n* = 8–10). **(A)** Fasting blood glucose. **(B,C)** GTT and the AUC for the GTT. **(D,E)** ITT and the AUC for the ITT. **(F)** Food intake. **(G)** Physical activity. **(H)** Absorbed energy. **(I)** Energy intake. **(J)** No absorbed energy. **(K–N)** Western blotting analysis of GLUT4 expression in WAT, BAT, Liver, and Muscle. **(O)** Western blotting analysis of inflammation-related proteins expression in WAT. **(P)** Immunohistochemical staining for GLUT4 in WAT. Magnification is 200×, Scale bar = 100 μm. *p < 0.05, **p < 0.01, and ***p < 0.001. NS, not statistically significant. The results are presented as mean ± SEM. HFD: High-fat diet; HFD + GEBE: High-fat diet with GEBE.

Four-week-old male C57BL/6 mice were fed an HFD, with or without GEBE for 12 weeks. The fasting blood glucose concentrations of the HFD mice were significantly reduced by GEBE treatment (Figure 1A). To assess the effect of GEBE on glucose and insulin tolerance, we performed GTT and ITT, and found that the administration of GEBE significantly improved both the glucose tolerance and insulin sensitivity of the mice, as assessed using the respective AUCs (Figures 1B–E). These effects of GEBE occurred without any differences in food intake, energy intake, or physical activity (Figures 1F–J).

We also analyzed GLUT4 protein expression by western blotting in the principal insulin target tissues and found that it was significantly higher in the WAT of GEBE-treated than in control HFD-fed mice, but that its expression in the BAT, liver, or muscle did not differ between the groups (Figures 1K–N). Similar results were obtained using immunohistochemical staining (Figure 1P). This suggests that GEBE increases the uptake of glucose by WAT, which may improve glucose metabolic status. We also found less inflammation in the WAT of GEBE-treated mice, as indicated by lower protein expression of TLR4 and NF-κB (Figure 1O). These results suggest that GEBE may improve the insulin sensitivity and glucose tolerance of HFD-fed mice by increasing GLUT4 expression in WAT and reducing inflammation.

### *Gastrodia elata* Blume extract administration alters the composition of the gut microbiota

**FIGURE 2:**
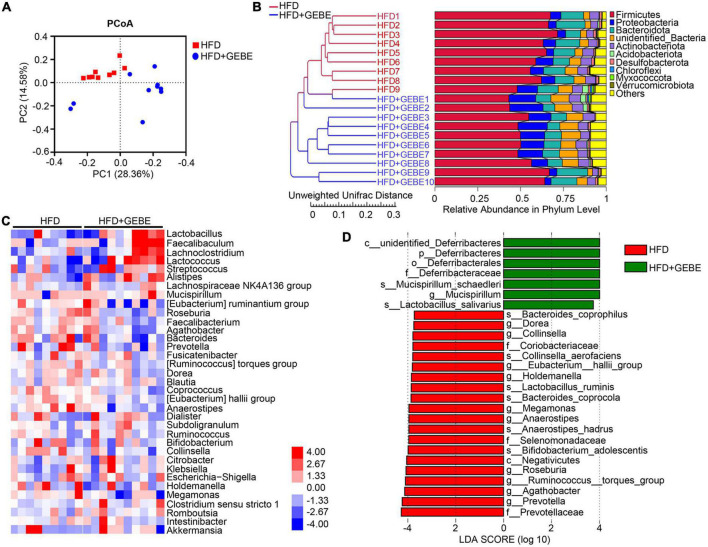
GEBE regulates the gut microbiota in HFD-induced C57BL/6 mice (*n* = 9–10). **(A)** Principal coordinate analysis (PCoA) based on unweighted UniFrac distances. **(B)** Unweighted UniFrac analysis using unweighted pair group method with arithmetic mean (UPGMA) clustering analysis. **(C)** Heatmap of gut microbiota at the genus level in mice. **(D)** Discriminative taxa determined by LEfSe between two groups (log10 LDA > 3.73). HFD: High-fat diet; HFD + GEBE: High-fat diet with GEBE.

Multiple recent studies have shown that alterations in the gut microbiota are associated with metabolic diseases, such as diabetes (Sun et al., 2018; Zhao et al., 2018; Li et al., 2020). Therefore, we collected feces from the mice for gut microbiological analysis. Principal coordinate analysis (PCoA), based on unweighted UniFrac distances, showed significant differences in the gut microbiota of GEBE-treated and control HFD-fed mice (Figure 2A). Interestingly, the results of unweighted UniFrac analysis using unweighted pair group method with arithmetic mean (UPGMA) clustering analysis showed that GEBE treatment increased the abundance of *Proteobacteria* and *unidentified Bacteria* at the phylum level (Figure 2B). Heat map analysis was used to identify the microbial genera that contributed to these differences in the gut microbial composition of the two experimental groups (Figure 2C). Of the top 35 most abundant genera identified, GEBE significantly increased the relative abundances of *Faecalibaculum*, *Lactobacillus*, and others. Linear discriminant analysis effect size (LEfSe) showed that GEBE treatment enriched the microbiota of the mice with respect to many species, including *Mucispirillum schaedleri* and *Lactobacillus salivarius*, vs. HFD-fed mice (Figure 2D). Taken together, these results demonstrate that GEBE affects the composition of the gut microbiota of HFD-fed mice.

### The gut microbiota contributes to the beneficial effects of *Gastrodia elata* Blume extract on glucose homeostasis

**FIGURE 3:**
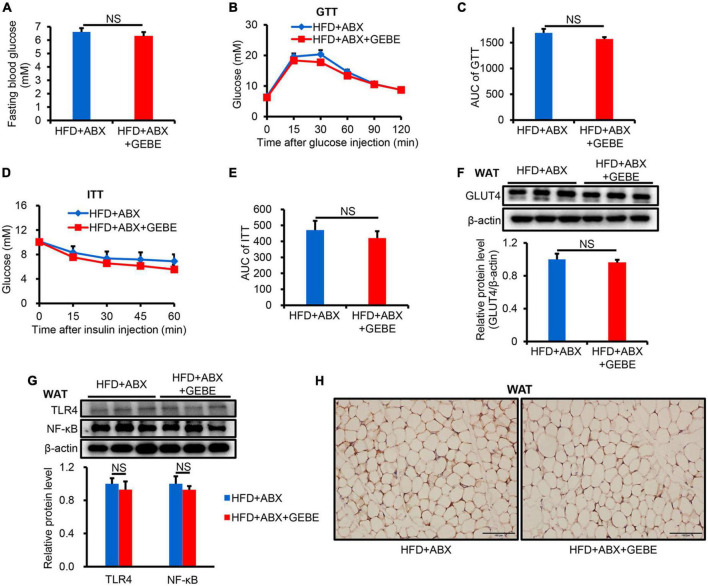
ABX treatment eliminates the beneficial effects of GEBE on HFD-induced C57BL/6 mice (*n* = 8–10). **(A)** Fasting blood glucose. **(B,C)** GTT and the AUC for the GTT. **(D,E)** ITT and the AUC for the ITT. **(F)** The protein levels of GLUT4 in WAT were measured by western blotting. **(G)** ABX eliminates the inhibitory effect of GEBE on the expression of inflammation-associated proteins in WAT. **(H)** Immunohistochemical staining for GLUT4 in WAT. Magnification is 200×, Scale bar = 100 μm. NS, not statistically significant. The results are presented as mean ± SEM. HFD + ABX: High-fat diet and water with combined antibiotics; HFD + ABX + GEBE: High-fat diet with GEBE, and water with combined antibiotics.

To investigate whether the gut microbiota mediates the beneficial effects of GEBE on the glucose and insulin tolerance of HFD-fed mice, we performed an antibiotic experiment. We found no difference in the fasting blood glucose concentrations of the two groups (Figure 3A). Meanwhile, GTT and ITT revealed that ABX treatment completely prevented the beneficial effects of GEBE on glucose tolerance and insulin sensitivity in HFD-fed mice (Figures 3B–E). There were no differences in the protein expression of GLUT4, TLR4, or NF-κB in the WAT of the two groups (Figures 3F–H). Thus, GEBE requires an intact microbiota to have its beneficial effects in HFD-fed mice.

**FIGURE 4:**
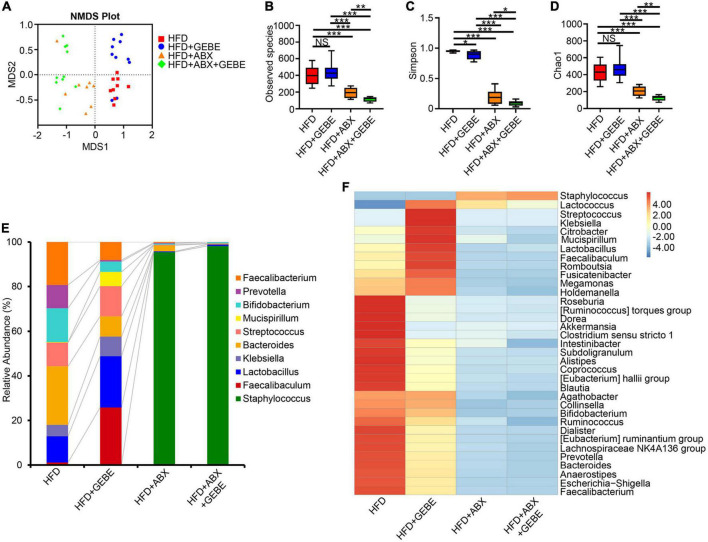
ABX treatment suppressed the intestinal microbiota in mice (*n* = 8–10). **(A)** Non-metric multi-dimensional scaling **(NMDS)** analysis. **(B–D)** The alpha diversity boxplots were measured using Observed species **(B)**, Chao1 **(C),** and Shannon **(D)** diversity indexes. **(E)** The relative abundance of bacteria at genus levels. **(F)** Heatmap of gut microbiota at the genus level. *p < 0.05, **p < 0.01, and ***p < 0.001. NS, not statistically significant. The results are presented as mean ± SEM. HFD, High-fat diet; HFD + GEBE, High-fat diet with GEBE; HFD + ABX, High-fat diet and water with combined antibiotics; HFD + ABX + GEBE, High-fat diet with GEBE, and water with combined antibiotics.

Interestingly, Non-Metric Multi-Dimensional Scaling (NMDS) analysis showed no differences in the gut microbiota of the mice in the HFD + ABX and HFD + ABX + GEBE groups, in contrast to the differences identified between the HFD and HFD + GEBE groups (Figure 4A). Analysis of the bacterial communities using the Observed species, Chao 1, and Shannon indices, which reflect α-diversity, revealed that the number of enterobacterial species, the diversity, and the homogeneity of the microbiota of the ABX-treated mice were significantly lower than those of the non-ABX-treated mice (Figures 4B–D). The relative abundance of bacterial taxa and a heatmap of the genera identified showed that ABX treatment significantly reduced the relative abundance of most bacterial genera, including *Faecalibaculum* and *Lactobacillus*. In addition, *Staphylococcus* was the most abundant genus among the ABX-treated mouse enterobacteria (Figures 4E,F). These data imply that the intestinal microbiota of the mice was disrupted and that the beneficial effects of GEBE were eliminated after ABX treatment. This further implies that the beneficial effects of GEBE on glucose and insulin tolerance may depend on the presence of a normal intestinal microbiota.

### *Gastrodia elata* Blume extract affects the serum bile acid profile of high-fat diet-fed mice

**FIGURE 5:**
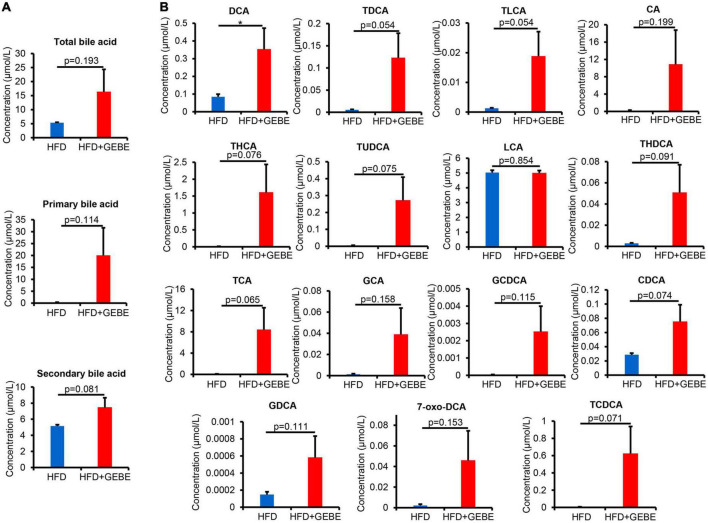
GEBE supplementation alters the serum bile acid profile in HFD-induced C57BL/6 mice (*n* = 8). **(A)** Serum levels of total bile acids, primary bile acids, and secondary bile acids in HFD-induced mice with GEBE treatment. **(B)** Bile acid levels in the serum. *p < 0.05. NS, not statistically significant. The results are presented as mean ± SEM. HFD, High-fat diet; HFD + GEBE, High-fat diet with GEBE.

The gut microbiota is involved in bile acid metabolism, and bile acids have effects on glucose metabolism (Jiang et al., 2022). To determine whether the effects of GEBE on the gut microbiota translated into changes in bile acid profile, we profiled the bile acid composition of the mouse serum. We found higher serum concentrations of bile acids in GEBE-treated mice, with the concentrations of secondary bile acids tending to be higher than those of primary bile acids (Figure 5A). In particular, the concentration of deoxycholic acid (DCA), a secondary bile acid, was significantly increased by GEBE, and those of both taurodeoxycholic acid (TDCA) and taurolithocholic acid (TLCA) also tended to be higher (Figure 5B).

**FIGURE 6:**
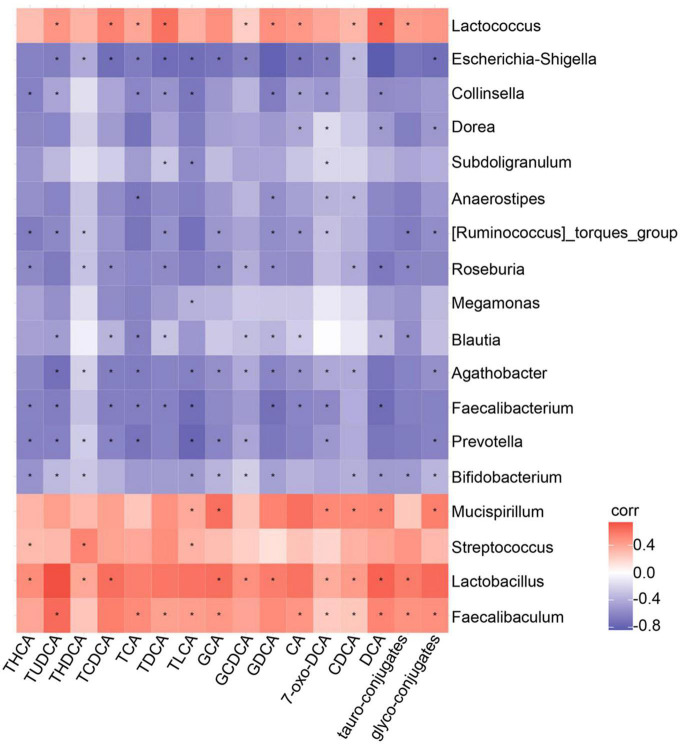
Assessment of correlation between gut microbiota and serum bile acid profile in HFD-induced mice treated with GEBE: Spearman correlations between the relative abundance of bacterial genera and bile acid profile in the serum (*n* = 8). *p < 0.05.

To investigate the relationships between circulating metabolite concentrations and the composition of the gut microbiota in the groups, we performed Spearman correlation analysis (Figure 6). We found strong positive correlations between the abundances of *Lactobacillus*, *Faecalibaculum*, *Mucispirillum*, and *Lactococcus* and the serum DCA concentration. Additionally, there were also positive correlations between the abundances of *Faecalibaculum*, *Lactococcus*, and the serum TDCA concentration. Finally, the TLCA concentration positively correlated with the abundances of *Lactobacillus*, *Faecalibaculum*, and *Mucispirillum* (Figure 6). Taken together, these data indicate that GEBE changes the host serum concentrations of bile acids, which are metabolites of the intestinal microbiota.

## Discussion

**FIGURE 7:**
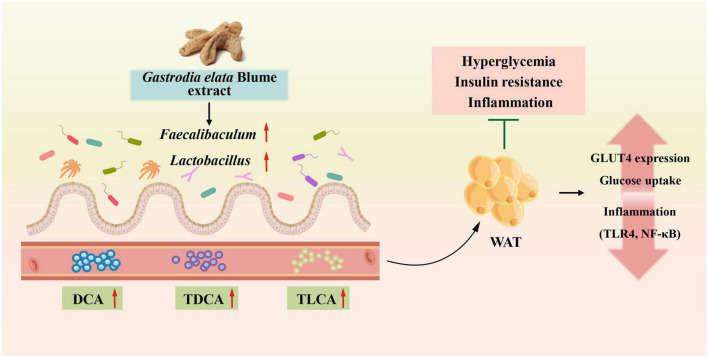
GEBE ameliorates T2D by increasing GLUT4 expression in WAT, remodeling the gut microbiota, and modifying serum bile acid concentrations.

In the present study, we have shown that GEBE ameliorates the glucose intolerance and insulin resistance of HFD-fed mice. We have also shown that GEBE increases GLUT4 expression in WAT, alters the composition of the intestinal flora, and alters the host serum bile acid profile, all of which may explain its metabolic effects (Figure 7).

A growing number of studies have shown that TCM has an ameliorative effect on metabolic diseases because of its ability to regulate the gut microbiota. *Hirsutella sinensis* may reduce diet-induced obesity and metabolic disorders by increasing levels of *Parabacteroides goldsteinii* in the gut, improving intestinal integrity, reducing metabolic endotoxemia, inflammation, insulin resistance, and inducing thermogenesis (Wu et al., 2019). Polysaccharide components in herbs have been reported to regulate the intestinal microbial structure (Wu et al., 2019; Luo et al., 2022). And there is also 30% polysaccharide in GEBE, which changed the structure of intestinal flora in HFD-induced mice. In the present study, we found that the abundances of the genera *Faecalibaculum*, *Lactobacillus*, and *Mucispirillum* in HFD-fed mice were significantly affected by GEBE administration (Figure 2C). Consistent with this, a previous study showed that GEBE increased the abundance of *Faecalibaculum* and thereby ameliorated early atherosclerosis in mice (Liu et al., 2021). In addition, the abundance of *Faecalibaculum* was previously reported to be closely and negatively associated with characteristics of the metabolic syndrome (Wu et al., 2022a). *Lactobacillus*, a recognized probiotic, ameliorates glucose metabolism disorders and disturbances of the intestinal ecology in a rat model of T2D and reduces inflammation and the circulating concentration of lipopolysaccharide (El-Baz et al., 2021; Wu et al., 2022b). We also found that GEBE treatment increases the abundances of *Lactobacillus salivarius* and *Mucispirillum schaedleri* by means of LEfSe (Figure 2D). In a recent study, *Lactobacillus salivarius* influenced glucose metabolism in diabetic mice by increasing the expression of glucose transporter protein 2 in human intestinal epithelial cells (Hsieh et al., 2020; Wang et al., 2022). Furthermore, *Mucispirillum schaedleri* has a protective effect against *Salmonella-*induced colitis in mice (Herp et al., 2019). Further, we used antibiotic treatment to eliminate the intestinal microbiota of mice and found that the beneficial effects of GEBE were abrogated, which implies that GEBE achieves these effects by modulating the intestinal flora.

Bile acids are major metabolites of the gut microbiota and affect a variety of host physiological processes (Sun et al., 2018; Qi et al., 2019; Li et al., 2020). In the present study, the serum concentrations of secondary bile acids were increased by GEBE more substantially than those of primary bile acids. The composition of the intestinal microbiota influences host physiology primarily through the production of secondary, rather than primary, bile acids (Sun et al., 2018; Qi et al., 2019). DCA, a secondary bile acid that was present in a significantly high circulating concentration in GEBE-treated mice in the present study, is an endogenous ligand and activator of TGR5 (Katsuma et al., 2005). It has previously been reported that DCA can activate TGR5 and thus regulate host glucose metabolism (Li et al., 2020). In addition, TDCA and TLCA, which are agonists of TGR5 and FXR (Ding et al., 2015), were present at higher concentrations following GEBE treatment. TDCA is one of the most potent natural inducers of GLP-1 secretion yet identified, and its rectal administration improves glucose homeostasis in patients with obesity and T2D (Brighton et al., 2015). Previous studies show that the intravenous administration of TLCA to mice causes beiging in the WAT, and increases fat oxidation and energy expenditure (Eggink et al., 2018).

We also found an association between the serum bile acid profile and the intestinal microbiota by Spearman correlation analysis (Figure 6). In particular, DCA showed close positive correlations with the abundances of *Lactobacillus*, *Faecalibaculum*, *Mucispirillum*, and *Lactococcus*. It has also been reported that *Lactobacillus* expresses hydroxysteroid dehydrogenase, which converts primary bile acids to secondary bile acids by 7-dehydroxylation, and generates DCA in this way (Chen et al., 2019). Moreover, we found close associations of the circulating concentrations of TLCA and TDCA with the abundances of *Faecalibaculum*, *Lactococcus*, and other intestinal microbes. We found that GEBE increased the abundances of specific intestinal taxa, including *Lactobacillus* and *Faecalibaculum*, and also affected the concentrations of secondary bile acids, such as DCA, which may explain its effects on glucose tolerance. Cells utilize membrane glucose transporters for glucose uptake, and GLUT4 is the insulin-responsive transporter and is highly expressed in adipose tissue, muscle, and liver (Deng and Yan, 2016; Yuan et al., 2022). Low GLUT4 expression and a disruption of glucose transport are thought to be involved in the mechanism of the impaired glucose metabolism that characterizes T2D (Deng and Yan, 2016). In the present study, GEBE was found to increase the expression of GLUT4 in WAT, but not in other insulin target tissues. Previous *in vitro* experiments have shown that bile acids induce *Glut4* transcription in 3T3-L1 and HepG2 cells (Shen et al., 2008), and bile acids also increase GLUT4 expression in Zucker (fa/fa) rats, thereby ameliorating insulin resistance (Cipriani et al., 2010). We also found that the expression of TLR4 and NF-κB was lower in WAT following GEBE treatment. It has previously been reported that the TLR4-NF-κB signaling pathway is involved in the development of T2D. The activation of this pathway promotes insulin resistance, and therefore its downregulation may be associated with improved insulin action (Saad et al., 2016; Mukhuty et al., 2021). Interestingly, it has also been reported that the administration of bile acids inhibits the TLR4-NF-κB signaling pathway, and thereby reduces inflammation and insulin resistance (Chen et al., 2019; Xing et al., 2020; Liu et al., 2022).

## Conclusion

In conclusion, the present findings suggest that GEBE may ameliorate HFD-induced T2D through effects on the gut microbiota, host bile acid profile, and GLUT4 expression in WAT. In addition, we have provided evidence that an intact gut microbiota is required for the beneficial effects of GEBE. These findings provide potential molecular mechanisms for the beneficial effects of GEB in T2D.

## Data availability statement

The datasets presented in this study can be found in online repositories. The names of the repository/repositories and accession number(s) can be found in the article/Supplementary material.

## Ethics statement

This animal study was reviewed and approved by the Animal Ethics Committee of Yanbian University (SYXK2020-0009).

## Author contributions

DW, J-XW, and CY: methodology, investigation, data curation, visualization, formal analysis, and writing—original draft. YL, HL, and DL: investigation and data curation. JZ, Z-BL, and S-ZH: validation and data curation. Z-YJ, S-YC, and L-HY: formal analysis. J-DK: supervision, resources, and writing—review and editing. L-HQ: conceptualization, supervision, funding acquisition, and writing—review and editing. All authors contributed to the article and approved the submitted version.

## References

[B1] Bai Y. Mo K. Wang G. Chen W. Zhang W. Guo Y. (2021). Intervention of gastrodin in Type 2 diabetes mellitus and its mechanism. *Front. Pharmacol.* 12:710722. 10.3389/fphar.2021.710722 34603025 PMC8481818

[B2] Brighton C. A. Rievaj J. Kuhre R. E. Glass L. L. Schoonjans K. Holst J. J. (2015). Bile acids trigger GLP-1 release predominantly by accessing basolaterally located G protein-coupled bile acid receptors. *Endocrinology* 156 3961–3970. 10.1210/en.2015-1321 26280129 PMC4606749

[B3] Chang C. J. Lin C. S. Lu C. C. Martel J. Ko Y. F. Ojcius D. M. (2015). Ganoderma lucidum reduces obesity in mice by modulating the composition of the gut microbiota. *Nat. Commun.* 6:7489. 10.1038/ncomms8489 26102296 PMC4557287

[B4] Chen M. L. Takeda K. Sundrud M. S. (2019). Emerging roles of bile acids in mucosal immunity and inflammation. *Mucosal Immunol.* 12 851–861. 10.1038/s41385-019-0162-4 30952999

[B5] Cipriani S. Mencarelli A. Palladino G. Fiorucci S. (2010). FXR activation reverses insulin resistance and lipid abnormalities and protects against liver steatosis in Zucker (fa/fa) obese rats. *J. Lipid Res.* 51 771–784. 10.1194/jlr.M001602 19783811 PMC2842143

[B6] de Vos W. M. Tilg H. Van Hul M. Cani P. D. (2022). Gut microbiome and health: mechanistic insights. *Gut* 71 1020–1032. 10.1136/gutjnl-2021-326789 35105664 PMC8995832

[B7] Deng D. Yan N. (2016). GLUT, SGLT, and SWEET: structural and mechanistic investigations of the glucose transporters. *Protein Sci.* 25 546–558. 10.1002/pro.2858 26650681 PMC4815417

[B8] Ding L. Yang L. Wang Z. Huang W. (2015). Bile acid nuclear receptor FXR and digestive system diseases. *Acta Pharm. Sin. B* 5 135–144. 10.1016/j.apsb.2015.01.004 26579439 PMC4629217

[B9] Eggink H. M. Tambyrajah L. L. van den Berg R. Mol I. M. van den Heuvel J. K. Koehorst M. (2018). Chronic infusion of taurolithocholate into the brain increases fat oxidation in mice. *J. Endocrinol.* 236 85–97. 10.1530/joe-17-0503 29233934

[B10] El-Baz A. M. Shata A. Hassan H. M. El-Sokkary M. M. A. Khodir A. E. (2021). The therapeutic role of *Lactobacillus* and montelukast in combination with metformin in diabetes mellitus complications through modulation of gut microbiota and suppression of oxidative stress. *Int. Immunopharmacol.* 96:107757. 10.1016/j.intimp.2021.107757 33991997

[B11] Herp S. Brugiroux S. Garzetti D. Ring D. Jochum L. M. Beutler M. (2019). *Mucispirillum schaedleri* antagonizes salmonella virulence to protect mice against colitis. *Cell Host Microbe* 25 681–694.e8. 10.1016/j.chom.2019.03.004. 31006637

[B12] Hsieh P. S. Ho H. H. Hsieh S. H. Kuo Y. W. Tseng H. Y. Kao H. F. (2020). *Lactobacillus salivarius* AP-32 and *Lactobacillus reuteri* GL-104 decrease glycemic levels and attenuate diabetes-mediated liver and kidney injury in db/db mice. *BMJ Open Diab. Res. Care* 8:e001028. 10.1136/bmjdrc-2019-001028 32332068 PMC7202753

[B13] Huang Y. J. Choong L. C. Panyod S. Lin Y. E. Huang H. S. Lu K. H. (2021). *Gastrodia elata* Blume water extract modulates neurotransmitters and alters the gut microbiota in a mild social defeat stress-induced depression mouse model. *Phytother. Res.* 35 5133–5142. 10.1002/ptr.7091 34327733

[B14] Huo J. Lei M. Li F. Hou J. Zhang Z. Long H. (2021). Structural characterization of a polysaccharide from gastrodia elata and its bioactivity on gut microbiota. *Molecules* 26:4443. 10.3390/molecules26154443 34361604 PMC8348156

[B15] Jiang X. Sun B. Zhou Z. (2022). Preclinical studies of natural products targeting the gut microbiota: beneficial effects on diabetes. *J. Agric Food Chem.* 70 8569–8581. 10.1021/acs.jafc.2c02960 35816090

[B16] Katsuma S. Hirasawa A. Tsujimoto G. (2005). Bile acids promote glucagon-like peptide-1 secretion through TGR5 in a murine enteroendocrine cell line STC-1. *Biochem. Biophys. Res. Commun.* 329 386–390. 10.1016/j.bbrc.2005.01.139 15721318

[B17] Li M. Zhou W. Dang Y. Li C. Ji G. Zhang L. (2020). Berberine compounds improves hyperglycemia via microbiome mediated colonic TGR5-GLP pathway in db/db mice. *Biomed. Pharmacother.* 132:110953. 10.1016/j.biopha.2020.110953 33254441

[B18] Liu F. Y. Wen J. Hou J. Zhang S. Q. Sun C. B. Zhou L. C. (2021). Gastrodia remodels intestinal microflora to suppress inflammation in mice with early atherosclerosis. *Int. Immunopharmacol.* 96:107758. 10.1016/j.intimp.2021.107758 34162137

[B19] Liu H. Wang J. Ding Y. Shi X. Ren H. (2022). Antibiotic pretreatment attenuates liver ischemia-reperfusion injury by Farnesoid X receptor activation. *Cell Death Dis.* 13:484. 10.1038/s41419-022-04955-x 35597796 PMC9124217

[B20] Lu K. H. Ou G. L. Chang H. P. Chen W. C. Liu S. H. Sheen L. Y. (2020). Safety evaluation of water extract of Gastrodia elata Blume: genotoxicity and 28-day oral toxicity studies. *Regul. Toxicol. Pharmacol.* 114:104657. 10.1016/j.yrtph.2020.104657 32278877

[B21] Luo Y. Fang Q. Lai Y. Lei H. Zhang D. Niu H. (2022). Polysaccharides from the leaves of *Polygonatum sibiricum* red. regulate the gut microbiota and affect the production of short-chain fatty acids in mice. *AMB Express* 12:35. 10.1186/s13568-022-01376-z 35312878 PMC8938542

[B22] Luo Z. B. Luo Q. R. Xuan M. F. Han S. Z. Wang J. X. Guo Q. (2019). Comparison of internal organs between myostatin mutant and wild-type piglets. *J. Sci. Food Agric.* 99 6788–6795. 10.1002/jsfa.9962 31368537

[B23] Minokoshi Y. Alquier T. Furukawa N. Kim Y. B. Lee A. Xue B. (2004). AMP-kinase regulates food intake by responding to hormonal and nutrient signals in the hypothalamus. *Nature* 428 569–574. 10.1038/nature02440 15058305

[B24] Mukhuty A. Fouzder C. Kundu R. (2021). Blocking TLR4-NF-κB pathway protects mouse islets from the combinatorial impact of high fat and fetuin-A mediated dysfunction and restores ability for insulin secretion. *Mol. Cell Endocrinol.* 532:111314. 10.1016/j.mce.2021.111314 33989718

[B25] Nam Y. Yoon S. Baek J. Kim J. H. Park M. Hwang K. (2022). Heat-Killed *Lactiplantibacillus plantarum* LRCC5314 mitigates the effects of stress-related Type 2 diabetes in mice via gut microbiome modulation. *J. Microbiol. Biotechnol.* 32 324–332. 10.4014/jmb.2111.11008 34949748 PMC9628852

[B26] Ng C. F. Ko C. H. Koon C. M. Chin W. C. Kwong H. C. Lo A. W. (2016). The aqueous extract of rhizome of *Gastrodia elata Blume attenuates* locomotor defect and inflammation after traumatic brain injury in rats. *J. Ethnopharmacol.* 185 87–95. 10.1016/j.jep.2016.03.018 26979339

[B27] Patrone C. Eriksson O. Lindholm D. (2014). Diabetes drugs and neurological disorders: new views and therapeutic possibilities. *Lancet Diab. Endocrinol.* 2 256–262. 10.1016/s2213-8587(13)70125-6 24622756

[B28] Qi X. Yun C. Sun L. Xia J. Wu Q. Wang Y. (2019). Gut microbiota-bile acid-interleukin-22 axis orchestrates polycystic ovary syndrome. *Nat. Med.* 25 1225–1233. 10.1038/s41591-019-0509-0 31332392 PMC7376369

[B29] Quan L. H. Zhang C. Dong M. Jiang J. Xu H. Yan C. (2020). Myristoleic acid produced by enterococci reduces obesity through brown adipose tissue activation. *Gut* 69 1239–1247. 10.1136/gutjnl-2019-319114 31744910

[B30] Rozenberg K. Rosenzweig T. (2018). Sarcopoterium spinosum extract improved insulin sensitivity in mice models of glucose intolerance and diabetes. *PLoS One* 13:e0196736. 10.1371/journal.pone.0196736 29768504 PMC5955592

[B31] Saad M. J. Santos A. Prada P. O. (2016). Linking gut microbiota and inflammation to obesity and insulin resistance. *Physiology* 31 283–293. 10.1152/physiol.00041.2015 27252163

[B32] Shen H. Zhang Y. Ding H. Wang X. Chen L. Jiang H. (2008). Farnesoid X receptor induces GLUT4 expression through FXR response element in the GLUT4 promoter. *Cell Physiol. Biochem.* 22 1–14. 10.1159/000149779 18769028

[B33] Shibib L. Al-Qaisi M. Ahmed A. Miras A. D. Nott D. Pelling M. (2022). Reversal and remission of T2DM - an update for practitioners. *Vasc. Health Risk Manag.* 18 417–443. 10.2147/vhrm.S345810 35726218 PMC9206440

[B34] Sun L. Xie C. Wang G. Wu Y. Wu Q. Wang X. (2018). Gut microbiota and intestinal FXR mediate the clinical benefits of metformin. *Nat. Med.* 24 1919–1929. 10.1038/s41591-018-0222-4 30397356 PMC6479226

[B35] Sun X. Zhao H. Liu Z. Sun X. Zhang D. Wang S. (2020). Modulation of gut microbiota by fucoxanthin during alleviation of obesity in high-fat diet-fed mice. *J. Agric. Food Chem.* 68 5118–5128. 10.1021/acs.jafc.0c01467 32309947

[B36] Wahlström A. Sayin S. I. Marschall H. U. Bäckhed F. (2016). Intestinal crosstalk between bile acids and microbiota and its impact on host metabolism. *Cell Metab.* 24 41–50. 10.1016/j.cmet.2016.05.005 27320064

[B37] Wang C. H. Yen H. R. Lu W. L. Ho H. H. Lin W. Y. Kuo Y. W. (2022). Adjuvant probiotics of *Lactobacillus salivarius* subsp. salicinius AP-32, *L. johnsonii* MH-68, and *Bifidobacterium animalis* subsp. lactis CP-9 attenuate glycemic levels and inflammatory cytokines in patients with Type 1 diabetes mellitus. *Front. Endocrinol.* 13:754401. 10.3389/fendo.2022.754401 35299968 PMC8921459

[B38] Wu G. Sun X. Cheng H. Xu S. Li D. Xie Z. (2022a). Large yellow tea extract ameliorates metabolic syndrome by suppressing lipogenesis through SIRT6/SREBP1 pathway and modulating microbiota in leptin receptor knockout rats. *Foods* 11:1638. 10.3390/foods11111638 35681388 PMC9180543

[B39] Wu R. Zhou L. Chen Y. Ding X. Liu Y. Tong B. (2022b). Sesquiterpene glycoside isolated from loquat leaf targets gut microbiota to prevent type 2 diabetes mellitus in db/db mice. *Food Funct.* 13 1519–1534. 10.1039/d1fo03646g 35072186

[B40] Wu T. R. Lin C. S. Chang C. J. Lin T. L. Martel J. Ko Y. F. (2019). Gut commensal *Parabacteroides goldsteinii* plays a predominant role in the anti-obesity effects of polysaccharides isolated from *Hirsutella sinensis*. *Gut* 68 248–262. 10.1136/gutjnl-2017-315458 30007918

[B41] Xia T. Liu C. S. Hu Y. N. Luo Z. Y. Chen F. L. Yuan L. X. (2021). Coix seed polysaccharides alleviate type 2 diabetes mellitus via gut microbiota-derived short-chain fatty acids activation of IGF1/PI3K/AKT signaling. *Food Res. Int.* 150(Pt A):110717. 10.1016/j.foodres.2021.110717 34865748

[B42] Xing Y. Yan J. Niu Y. (2020). PXR: a center of transcriptional regulation in cancer. *Acta Pharm. Sin B* 10 197–206. 10.1016/j.apsb.2019.06.012 32082968 PMC7016272

[B43] Yuan Y. Kong F. Xu H. Zhu A. Yan N. Yan C. (2022). Cryo-EM structure of human glucose transporter GLUT4. *Nat. Commun.* 13:2671. 10.1038/s41467-022-30235-5 35562357 PMC9106701

[B44] Zhang H. Y. Tian J. X. Lian F. M. Li M. Liu W. K. Zhen Z. (2021). Therapeutic mechanisms of traditional Chinese medicine to improve metabolic diseases via the gut microbiota. *Biomed. Pharmacother.* 133:110857. 10.1016/j.biopha.2020.110857 33197760

[B45] Zhao L. Zhang F. Ding X. Wu G. Lam Y. Y. Wang X. (2018). Gut bacteria selectively promoted by dietary fibers alleviate type 2 diabetes. *Science* 359 1151–1156. 10.1126/science.aao5774 29590046

[B46] Zhou K. Wang J. Xie G. Zhou Y. Yan W. Pan W. (2015). Distinct plasma bile acid profiles of biliary atresia and neonatal hepatitis syndrome. *J. Proteome Res.* 14 4844–4850. 10.1021/acs.jproteome.5b00676 26449593

