# Supplementary material for: Gastrodia elata Blume extract improves high-fat diet-induced type 2 diabetes by regulating gut microbiota and bile acid profile
Source: Front Microbiol. 2022 Dec 2;13:1091712. doi: 10.3389/fmicb.2022.1091712 (PMC9756436; doi:10.3389/fmicb.2022.1091712)
Supplement: Supplementary file 1 [file Data_Sheet_1.PDF]

Supplementary Material

1 Supplementary Data

The raw reads of 16s rRNA gene sequences have been submitted to the NCBI BioSample database (Mice data: PRJNA898680). All sample metadata and intermediate analysis files are available at https://data.mendeley.com/datasets/przhxygkkm.

2 Supplementary Figures

Supplementary Figure 1

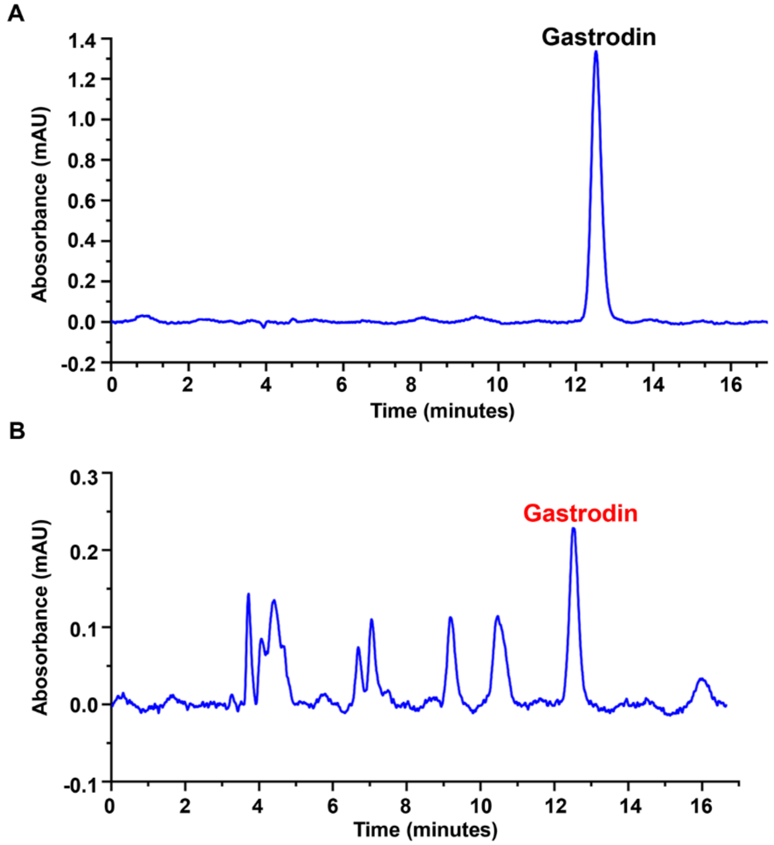

Supplementary Figure 1. HPLC result on gastrodin standard and GEBE. **(A)** HPLC profile of gastrodin standard (20 µg/mL). **(B)** HPLC profile of GEBE extract (1mg/mL).
